# Clonal diversity of *Acinetobacter baumannii* clinical isolates revealed by a snapshot study

**DOI:** 10.1186/1471-2180-13-234

**Published:** 2013-10-22

**Authors:** Xiaohui Wang, Fu Qiao, Rujia Yu, Yanyu Gao, Zhiyong Zong

**Affiliations:** 1 Center of Infectious Diseases, West China Hospital, Sichuan University, Guoxuexiang 37, Chengdu, China; 2 Division of Infectious Diseases, State Key Laboratory of Biotherapy, Chengdu, China; 3 Department of Infection Control, West China Hospital, Sichuan University, Chengdu, China

**Keywords:** Acinetobacter baumannii, Clonal relatedness, PFGE, MLST, bla_OXA-23_

## Abstract

**Background:**

*Acinetobacter baumannii* is a notorious opportunistic pathogen mainly associated with hospital-acquired infections. Studies on the clonal relatedness of isolates could lay the foundation for effective infection control. A snapshot study was performed to investigate the clonal relatedness of *A*. *baumannii* clinical isolates in our local settings.

**Results:**

Among 82 non-repetitive *Acinetobacter* spp. clinical isolates that were recovered during a period of four days in 13 hospitals in Sichuan, Southwest China, 67 isolates were identified as *A. baumannii*. Half of the 67 *A. baumannii* isolates were non-susceptible to carbapenems. *bla*_OXA-23_ was the only acquired carbapenemase gene detected, present in 40 isolates including five carbapenem-susceptible ones. The isolates belonged to 62 pulsotypes determined by PFGE and 31 sequence types (ST) by multi-locus sequence typing. Forty-three isolates belonged to the globally-disseminated clonal complex 92, among which ST75, ST92 and ST208 were the most common sequence types.

**Conclusions:**

Clinical isolates of *A. baumannii* were diverse in clonality in this snapshot study. However, most of the isolates belonged to the globally-distributed clonal complex CC92. ST75, ST92 and ST208 were the most common types in our region. In particular, ST208 might be an emerging lineage carrying *bla*_OXA-23_.

## Background

*Acinetobacter baumannii* is one of the common bacterial species responsible for hospital-acquired infections (HAIs) [1]. The prevalence of multi-drug resistant (MDR) *A. baumannii* in hospitals has been increasing worldwide [2], representing a serious challenge for clinical management and public health. Investigation on the clonal relatedness of *A. baumannii* in local settings could generate useful data to understand the local epidemiology of this opportunistic pathogen and therefore lay a foundation for an effective infection control program. Previous studies have focused on the clonal relatedness of *A. baumannii* but the vast majority of these studies were retrospective and used a collection of isolates either from outbreaks or with little information on their representativeness. For hospitals in Sichuan, Southwest China, *A. baumannii* was a huge problem as it was the most common bacterial species associated with HAIs and accounted for 17.3% of putative pathogens causing HAIs in a point prevalence survey [3]. Outbreaks due to *A. baumannii* had also been reported in our hospitals [4]. A snapshot study was therefore performed to investigate the clonal relatedness of *A. baumannii* clinical isolates in our local settings.

## Results and discussion

Among 82 non-repetitive isolates that were recovered from clinical specimens from June 22 to June 25, 2011 in 13 hospitals in Sichuan and were putatively identified as *A. baumannii* by automated microbiology systems, 67 isolates were validated to be *A. baumannii*. The vast majority (61/67, 91%) of the *A. baumannii* isolates were recovered from sputa or respiratory tract secretions. The remaining six isolates were from ascites, cerebrospinal fluid, drainage, pleural fluid or wound secretions. As for the clinical significance, *A. baumannii* isolates were considered as either colonizers (55.2%) or pathogens (44.8%) causing clinical infections.

About half of the *A. baumannii* isolates (35/67, 52.2%) were non-susceptible to carbapenems (34 non-susceptible to both imipenem and meropenem and 1 non-susceptible to meropenem only), which was in consistence with the 53% carbapenem resistance rate of *A. baumannii* in the 2010 report of Chinese Ministry of Health National Antimicrobial Resistance Investigation Net (MOHNARIN) [5]. Many isolates were non-susceptible to sulbactam (35/67, 52.2%), ceftazidime (39/67, 58.2%), ciprofloxacin (43/67, 64.2%) or cotrimoxazole (47/67, 70.1%) while all isolates were susceptible to polymyxin and rifampicin and only one isolate was non-susceptible to minocycline.

*bla*_OXA-23_ was the only acquired carbapenemase gene that was detected. Interestingly, it was present in 35/35 carbapenem-non-susceptible and 5/32 carbapenem-susceptible isolates. *bla*_OXA-23_ has been the most common carbapenemase gene in China, as a previous study reported that 322 out of 342 (94.2%) imipenem-non-susceptible *A. baumannii* isolates collected from 16 Chinese cities had *bla*_OXA-23_[6]. Although *bla*_OXA-23_ encodes a carbapenemase, this gene has also been detected in carbapenem-susceptible isolates before [7].

**Figure 1:**
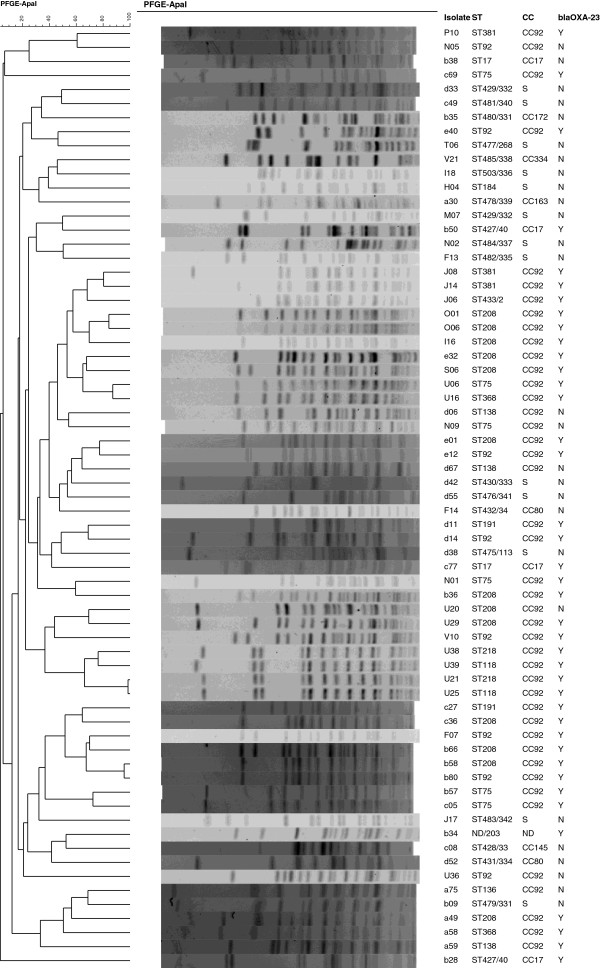
**PFGE patterns of *A. baumannii *isolates.** Dendrogram was generated by BioNumerics software with the unweighted pair-group method using arithmetic averages (UPGMA). Isolate name, ST, CC and the carriage of *bla*_OXA-23_ (Y, positive; N, negative) are indicated. The ST numbers shown after slash are assigned using the Pasteur MLST scheme.

**Figure 2:**
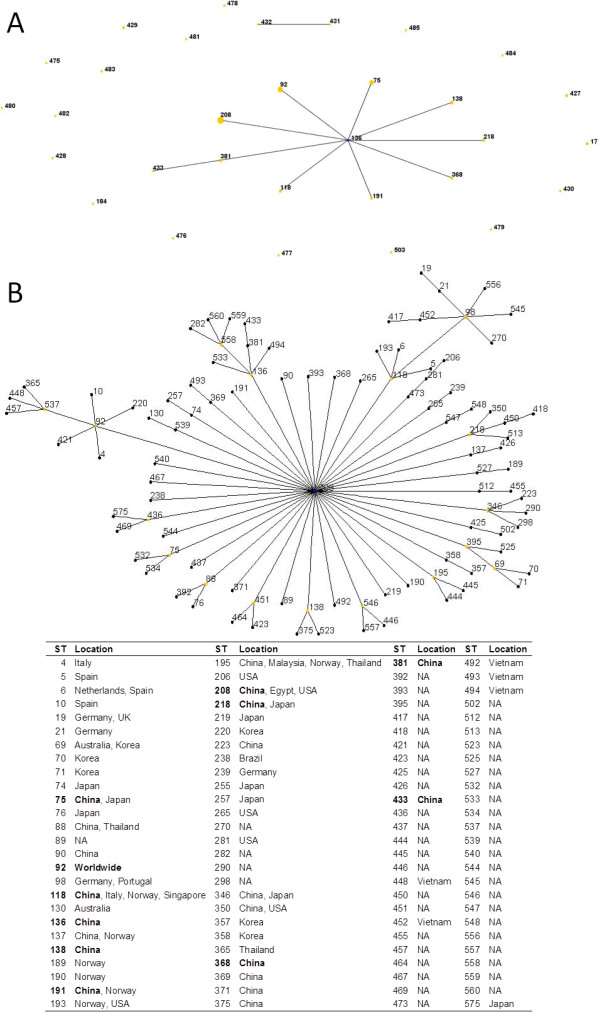
**STs seen in this study and the CC92. Panel A**. The relatedness of the STs seen in this study. **Panel B**. The clonal complex 92 (CC92). eBURST was performed using 6 as the minimum identical loci for the definition of CC and 3 as the minimum single locus variants. The countries of isolation are shown according to the pubmlst database. The STs found in this study are in bold. NA, information not available. ST208 is predicted as the founder ST for CC92 by eBURST.

The isolates were assigned to 62 pulsotypes determined by pulsed-field gel electrophoresis (PFGE), suggesting quite diverse clonal relatedness (Figure 1). A total of 31 sequence types (STs), including 19 new STs, were assigned for the isolates using the multi-locus sequence typing (MLST) with the pubmlst scheme (Table 1 and Figure 2). As the *gdhB* gene sequence was not obtained from isolate d34 despite repeated attempts using various primer pairs, the ST could not be assigned for this isolate. Of note, two isolates of the same pulsotypes were assigned to different STs, ST118 and ST218. However, ST118 and ST218 were found to be single locus variants to each other. This was in consistence with a previous study [8] reporting that isolates belonging to the same puslotype were not always of the same STs.

**Table 1:** **Profiles of *A. baumannii *** **clinical isolates**

**ST**^**1**^	**ST profile**^**1**^**:**	**CC**^**2**^	**Isolates no.**	**Hospital**^**3**^	**PFGE types**	**No., isolates carrying**	**No., carbapenem-non-susceptible isolates**
	***gltA*- *gyrB *- *gdhB *- *recA *- *cpn60*- *gpi *-*rpoD***						
						***bla***_**OXA-23**_	
17	1-12-12-11-4-10-3	17	2	WC	3, 36	1	0
75	1-3-3-2-2-11-3	92	6	DZ, MY, WC	4, 24, 26, 37, 50, 51	5	4
92	1-3-3-2-2-7-3	92	8	DY, DZ, MY, NC, WC	2, 8, 28, 34, 41, 47, 49, 56	6	6
118	1-3-3-2-2-3-3	92	2	MY	43, 44	2	2
136	1-3-3-2-2-16-3	92	1	WC	57	0	0
138	1-3-3-2-2-50-3	92	3	WC	25, 29, 61	1	1
184	36-31-59-28-4-54-4	S	1	SN	12	0	0
191	1-3-3-2-2-94-3	92	2	WC	33, 45	2	2
208	1-3-3-2-2-97-3	92	13	LS, LZ, MY, WC, YA	20 (2)^4^, 21, 22, 23, 27, 38, 39, 40, 46, 48, 49, 59	12	12
218	1-3-3-2-2-102-3	92	2	MY	42, 44	2	2
368	1-3-3-2-2-140-3	92	2	MY, WC	24, 60	2	2
381	1-81-3-3-2-2-16-3	92	3	LE, PZ	1, 18 (2)^4^	3	3
427/40	1-31-12-11-4-25-3	17/216	2	WC	15, 62	2	0
428/33	21-15-2-28-1-52-4	145/33	1	WC	54	0	0
429/332	1-34-56-1-4-144-45	S/216	2	MS, WC	5, 14	0	0
430/333	1-31-49-10-1-103-4	S/S	1	WC	30	0	0
431/334	34-12-68-41-1-104-4	80/34	1	WC	55	0	0
432/34	34-12-68-41-32-104-4	80/34	1	NC	32	0	0
433/2	1-81-3-1-2-16-3	92/2	1	PZ	19	1	1
475/113	18-44-2-28-1-151-71	S/113	1	WC	35	0	0
476/341	35-50-42-49-48-157-5	S/S	1	WC	31	0	0
477/268	1-50-56-2-1-152-26	S/216	1	ZG	9	0	0
478/339	33-89-40-26-32-91-5	163/S	1	WC	13	0	0
479/331	52-39-80-5-1-161-12	S/216	1	WC	58	0	0
480/331	1-34-80-28-1-160-45	172/216	1	WC	7	0	0
481/340	1-12-67-6-50-159-6	S/243	1	WC	6	0	0
482/335	1-90-6-1-36-102-6	S/216	1	NC	17	0	0
483/342	24-1-129-11-49-16-26	S/158	1	PZ	52	0	0
484/337	1-91-130-1-36-110-72	S/S	1	DZ	16	0	0
485/338	33-12-59-11-32-158-5	334/S	1	DY	10	0	0
503/336	1-62-80-28-35-164-45	S/216	1	LZ	11	0	0
ND/203	1-34-ND-28-1-165-45	/216	1	WC	53	1	0

^1^ND, not determined. For new STs (ST427 and above) and the undetermined ST by the pubmlst scheme, STs determined using the Pasteur MLST scheme are shown after the slash “/” with new STs being underlined.

^2^CC, clonal complex. S, singleton. CCs for STs determined using the Pasteur MLST scheme are shown after the slash “/” . The members of CCs for STs determined using the pubmlst scheme in Table 1 other than CC92 are listed as below. CC17 (n = 6): ST17, ST373, ST427, ST528, ST530, ST554; CC80 (n = 4): ST80, ST360, ST431, ST432; CC145 (n = 11): ST85, ST86, ST144, ST145, ST153, ST158, ST376, ST401, ST428, ST552, ST553; CC163 (n = 8): ST163, ST164, ST165, ST166, ST167, ST458, ST478, ST574; CC172 (n = 3): ST172, ST412, ST480; CC334 (n = 2): ST334, ST485.

^3^Hospital: DY, Deyang People’s Hospital, Deyang city; DZ, Dazhou Central Hospital, Dazhou city; LE, Leshan People’s Hospital, Leshan city; LS, The First Hospital of Liangshan Prefecture, Xichang city; LZ, Affiliated Hospital of Luzhou Medical College, Luzhou city; MS, Meishan People’s Hospital, Meishan City; MY, Mianyang Central Hospital, Mianyang city; NC, Affiliated Hospital of North Sichuan Medical College, Nanchong city; PZ, Panzhihua Central Hospital, Panzhihua city; SN, Suining Central Hospital, Suining city; WC, West China Hospital of Sichuan University, Chengdu city; YA, Yaan People’s Hospital,Yaan city; ZG, The First Municipal Hospital of Zigong, Zigong city.

^4^The number in brackets indicates the number of isolates belonging to the same pulsotype. Isolates of the same pulsotype were recovered from different patients in the same hospital.

ST75, ST92 and ST208 were the most common STs in our local settings as each of them were found in three to five hospitals scattered in different regions of the province. ST92 and ST75 were prevalent in West China Hospital in a study carried out in 2006 [7] and were also the most common STs in China in 2005 [9]. In contrast, ST208 had not been identified in China before, although it was found in five hospitals in this study. All our ST208 isolates except one carried *bla*_OXA-23_. ST208 has recently become one of the two most common STs of carbapenem-non-susceptible isolates in the United States [10], suggesting that ST208 might be an emerging lineage carrying carbapenem resistance determinants.

ST92, a globally-distributed type, and its 103 closely-related STs including ST75, ST118, ST136, ST138, ST191, ST208, ST218, ST368, ST381 and ST433 detected in this study comprised the clonal complex 92 (CC92, Figure 2), corresponding to the pan-European clone II (EUII; the international clone 2) [11]. Although diverse types were detected, 43 isolates belonged to the CC92. Among the 104 STs of CC92, 35 STs were different from ST92 only in the *gpi* locus. Isolate information was available for 22 of these 35 STs, 14 of which have only been found in the East or Southeast Asia at present. The 20 non-CC92 STs identified in the present study were either singletons (n = 12) or of a CC that was not a common international clone (Table 1).

A total of 575 STs were assigned in the *Acinetobacter* MLST database (http://pubmlst.org/abaumannii/, accessed by May 19, 2013), among which isolates identified as *A. baumannii* belonged to 545 STs. The 305-bp *gpi* locus of *A. baumannii* appeared to diverge much faster than other loci, with 149 *gpi* allele types comparing to 45 *gltA*, 84 *gyrB*, 85 *gdhB*, 53 *recA*, 42 *cpn60* and 63 *rpoD*. The occurrence of a different *gpi* allele for every three STs deposited in the pubmlst database raised the concern that *gpi* might not be an ideal locus for typing. A previous study [8] also suggested that *gpi* was not a good candidate for MLST analysis due to recombination. Therefore, the diversity of *A. baumannii* generated by variations in the *gpi* locus alone might need validation to appreciate whether the diversity is truly meaningful.

Isolates belonging to each of 19 new STs were assigned to 18 STs using the Pasteur scheme (Table 1). Among the 18 STs determined by the Pasteur scheme, 12 had not been seen before and therefore were truly new STs. Although 411 STs of *A. baumannii* had been assigned in the pubmlst database before this study, a few new STs were still detected in a relatively small collection, suggesting that *A. baumannii* is likely to be extremely diverse in clonal origins or is undergoing a significant clonal expansion. Of note, the isolates with new STs were all carbapenem-susceptible except one. *bla*_OXA-23_ was not detected in most (17/21) isolates of the novel STs. This phenomenon was also present in this study as all the local carbapenem-resistant isolates carrying *bla*_OXA-23_ belonged to CC92. It has been suggested that among carbapenem-resistant isolates some belonging to certain clonal complexes appeared to be more successful [12-14].

The diversity of *A. baumannii* isolates in our settings could provide useful information for infection control. The clonal diversity of *A. baumannii* and the fact that carbapenem resistance could be transmitted horizontally highlight that “horizontal” infection control measures such as environmental cleaning and hand hygiene should be reinforced to reduce the further spread of *A. baumannii*. Person-to-person transmission of carbapenem-non-susceptible *A. baumannii* carrying *bla*_OXA-23_ was indeed identified for several cases as evidenced by the fact that isolates recovered from different patients belonged to the same pulsotype (Table 1 and Figure 1). This suggests that effective infection control measures might need to include rapid identification of *bla*_OXA-23_ by molecular methods and also justifies contact precautions for patients with carbapenem-resistant isolates.

## Conclusions

This study provided a snapshot of *A. baumannii* population in clinical samples in our local settings. Significantly diverse clonal origins were identified but most isolates belonged to the globally-distributed CC92. Among CC92, ST75, ST92 and ST208 were the most common types in our region. The high prevalence of ST208 carrying *bla*_OXA-23_ suggests that ST208 appears to be an emerging lineage mediating the spread of carbapenem resistance. The diversity of *A. baumannii* suggested that the current MLST scheme might need to be further optimized and in particular the *gpi* gene might not be an ideal target for *Acinetobacter* MLST.

## Methods

### Strains

The study included all non-repetitive isolates (n = 82) that were recovered from clinical specimens from June 22 to June 25, 2011 in 13 hospitals in Sichuan, southwest China and were putatively identified as *A. baumannii* or belonging to the *Acinetobacter calcoaceticus-baumannii* complex using the Vitek II, MicroScan and Phoenix automated systems. The clinical samples were taken as part of standard patient care and therefore no ethical approval was applied for their use. The 13 hospitals are all tertiary with 19,051 beds in total (ranged from 800 to 4,300) including 3 university hospitals and 10 municipal ones. For each patient, only one isolate was collected. Genomic species identification was established by partially sequencing the *recA* gene as described previously [15].

### *In vitro* susceptibility test

MICs of meropenem, imipenem, ceftazidime, sulbactam, minocycline, polymyxin, ciprofloxacin, rifampicin and cotrimoxazole against *A. baumannii* isolates were determined using the agar dilution method following the recommendations of the Clinical and Laboratory Standards Institute (CLSI) [16]. MICs were interpreted according to the breakpoints established by CLSI [16], except for sulbactam and rifampicin, for which breakpoints from the French Society for Microbiology were used (for sulbactam, ≤8 mg/L for susceptible; for rifampicin, ≤8 μg/ml for susceptible and <16 mg/L for resistant) [17]. Resistance to imipenem or meropenem was defined as carbapenem resistance.

### Detection of carbapenemase-encoding genes

Genes encoding Class A carbapenemases (*bla*_GES_ and *bla*_KPC_), Class B metallo-β-lactamases (*bla*_IMP_, *bla*_VIM_, *bla*_SPM_, *bla*_GIM_, *bla*_SIM_ and *bla*_NDM_) or Class D OXA-type carbapenemases (*bla*_OXA-51_, *bla*_OXA-23_, *bla*_OXA-24_, *bla*_OXA-58_ and *bla*_OXA-143_) were screened as described previously [18-22]. Purified amplicons were sequenced in both directions using an ABI 3730 DNA analyzer (Applied Biosystems, Warrington, United Kingdom). Similarity searches were carried out using BLAST programs (http://www.ncbi.nlm.nih.gov/BLAST/).

### Strain typing

PFGE was employed to determine clonal relatedness of the isolates and was performed as described previously [12]. PFGE band patterns were analyzed using the BioNumerics software, version 6.6.4.0 (Applied Maths, St-Martens-Latem, Belgium). Pulsotypes were defined as isolates with PFGE band patterns of 80% similarity or above [23].

All *A. baumannii* isolates were subjected to MLST targeting seven housekeeping genes, *gltA*, *gyrB*, *gdhB*, *recA*, *cpn60*, *gpi* and *rpoD*[24]. As primers used previously were unable to amplify the *gdhB* and *gpi* alleles for some isolates [9,24,25], new primers were therefore designed for *gdhB* (gdhBxF1: ATTGGTTGCTGCCGAATAGT; gdhBxR1: TATGGGGGCCAGATAATCAA) and *gpi* (gpi-F2: AAAATCCATGCTGGGCAATA; gpi-R2: CCGAGTAATGCCATGAGAAC) genes [24]. New STs were deposited in the *Acinetobacter* MLST database (http://pubmlst.org/abaumannii/). eBURST (version 3, http://eburst.mlst.net/) was used to assign STs to CCs, which were defined for those sharing identical alleles at six of seven loci. CCs were named according to the number of the predicted founder ST except for CC92, which has been well defined in literature. If no founder ST was predicted by eBURST, the CC was named by the first ST assigned.

Isolates with new STs and isolate d34, of which ST could not be determined using the pubmlst scheme, were also subjected to MLST using the Pasteur scheme [26]. New STs determined using the Pasteur scheme have also been deposited into the database (http://www.pasteur.fr/mlst/Abaumannii.html).

## Competing interests

The authors declare that they have no competing interests.

## Authors’ contributions

WX carried out the molecular genetic studies, participated in the sequence alignment and drafted the manuscript. QF carried out the species identification. YR participated in the susceptibility tests. GY participated in the PCR. ZZ conceived of the study, and participated in its design and coordination and helped to draft the manuscript. All authors read and approved the final manuscript.

## References

[B1] Peleg AY Seifert H Paterson DL *Acinetobacter baumannii*: emergence of a successful pathogen Clin Microbiol Rev 2008 21 538 582 18625687 10.1128/CMR.00058-07 PMC2493088

[B2] Dijkshoorn L Nemec A Seifert H An increasing threat in hospitals: multidrug-resistant *Acinetobacter baumanni*i Nat Rev Microbiol 2007 5 939 951 18007677 10.1038/nrmicro1789

[B3] Zong Z Qiao F Yin W Xu S A large-scale survey on the point prevalence of healthcare-associated infections in southwest China IDWeek 2012 San Diego, CA: Poster Nr 1171

[B4] Zong Z Lu X Valenzuela JK Partridge SR Iredell J An outbreak of carbapenem-resistant *Acinetobacter baumannii* producing OXA-23 carbapenemase in western China Int J Antimicrob Agents 2008 31 50 54 18077141 10.1016/j.ijantimicag.2007.08.019

[B5] Li Y Lu Y Wang S Mohnarin report 2010: surveillance of antimicrobial resistance in nonfermenting gram-negative bacteria Chin J Nosocomiol 2011 21 5133 5137 (behalf of Mohnarin)

[B6] Zhou H Yang Q Yu YS Wei ZQ Li LJ Clonal spread of imipenem-resistant *Acinetobacter baumannii* among different cities of China J Clin Microbiol 2007 45 4054 4057 17942662 10.1128/JCM.00343-07 PMC2168573

[B7] Wang X Zong Z Lu X Tn*2008* is a major vehicle carrying *bla*_OXA-23_ in *Acinetobacter baumannii* from China Diagn Microbiol Infect Dis 2011 69 218 222 21251570 10.1016/j.diagmicrobio.2010.10.018

[B8] Hamouda A Evans BA Towner KJ Amyes SG Characterization of epidemiologically unrelated *Acinetobacter baumannii* isolates from four continents by use of multilocus sequence typing, pulsed-field gel electrophoresis, and sequence-based typing of *bla*_OXA-51_-like genes J Clin Microbiol 2010 48 2476 2483 20421437 10.1128/JCM.02431-09 PMC2897490

[B9] Fu Y Zhou J Zhou H Yang Q Wei Z Yu Y Li L Wide dissemination of OXA-23-producing carbapenem-resistant *Acinetobacter baumannii* clonal complex 22 in multiple cities of China J Antimicrob Chemother 2010 65 644 650 20154023 10.1093/jac/dkq027

[B10] Adams-Haduch JM Onuoha EO Bogdanovich T Tian GB Marschall J Urban CM Spellberg BJ Rhee D Halstead DC Pasculle AW Molecular epidemiology of carbapenem-nonsusceptible *Acinetobacter baumannii* in the United States J Clin Microbiol 2011 49 3849 3854 21918019 10.1128/JCM.00619-11 PMC3209126

[B11] Mugnier PD Poirel L Naas T Nordmann P Worldwide dissemination of the *bla*_OXA-23_ carbapenemase gene of *Acinetobacter baumannii* Emerg Infect Dis 2010 16 35 40 20031040 10.3201/eid1601.090852 PMC2874364

[B12] Seifert H Dolzani L Bressan R van der Reijden T Van Strijen B Stefanik D Heersma H Dijkshoorn L Standardization and interlaboratory reproducibility assessment of pulsed-field gel electrophoresis-generated fingerprints of *Acinetobacter baumannii* J Clin Microbiol 2005 43 4328 4335 16145073 10.1128/JCM.43.9.4328-4335.2005 PMC1234071

[B13] Karah N Sundsfjord A Towner K Samuelsen O Insights into the global molecular epidemiology of carbapenem non-susceptible clones of *Acinetobacter baumannii* Drug Resist Updat 2012 15 237 247 22841809 10.1016/j.drup.2012.06.001

[B14] Zarrilli R Pournaras S Giannouli M Tsakris A Global evolution of multidrug-resistant *Acinetobacter baumannii* clonal lineages Int J Antimicrob Agents 2013 41 11 19 23127486 10.1016/j.ijantimicag.2012.09.008

[B15] Krawczyk B Lewandowski K Kur J Comparative studies of the *Acinetobacter* genus and the species identification method based on the *recA* sequences Mol Cell Probes 2002 16 1 11 12005442 10.1006/mcpr.2001.0388

[B16] CLSI Performance Standards for Antimicrobial Susceptibility Testing; Twenty-First Informational Supplement 2011 Wayne, PA: The Clinical and Laboratory Standards Institute

[B17] Comite’de lAntibiogramme de la Socie’te’ Franc¸aise de Microbiologie Communique’ 2009 Paris, France: Socie´te´ Franc¸aise de Microbiologie

[B18] Woodford N Ellington MJ Coelho JM Turton JF Ward ME Brown S Amyes SG Livermore DM Multiplex PCR for genes encoding prevalent OXA carbapenemases in *Acinetobacter* spp Int J Antimicrob Agents 2006 27 351 353 16564159 10.1016/j.ijantimicag.2006.01.004

[B19] Higgins PG Lehmann M Seifert H Inclusion of OXA-143 primers in a multiplex polymerase chain reaction (PCR) for genes encoding prevalent OXA carbapenemases in *Acinetobacter* spp Int J Antimicrob Agents 2010 35 305 20022220 10.1016/j.ijantimicag.2009.10.014

[B20] Ellington MJ Kistler J Livermore DM Woodford N Multiplex PCR for rapid detection of genes encoding acquired metallo-β-lactamases J Antimicrob Chemother 2007 59 321 322 17185300 10.1093/jac/dkl481

[B21] Poirel L Le Thomas I Naas T Karim A Nordmann P Biochemical sequence analyses of GES-1, a novel class A extended-spectrum β-lactamase, and the class 1 integron In*52* from *Klebsiella pneumoniae* Antimicrob Agents Chemother 2000 44 622 632 10681329 10.1128/aac.44.3.622-632.2000 PMC89737

[B22] Bradford PA Bratu S Urban C Visalli M Mariano N Landman D Rahal JJ Brooks S Cebular S Quale J Emergence of carbapenem-resistant *Klebsiella* species possessing the class A carbapenem-hydrolyzing KPC-2 and inhibitor-resistant TEM-30 β-lactamases in New York City Clin Infect Dis 2004 39 55 60 15206053 10.1086/421495

[B23] Van Belkum A Tassios PT Dijkshoorn L Haeggman S Cookson B Fry NK Fussing V Green J Feil E Gerner-Smidt P Guidelines for the validation and application of typing methods for use in bacterial epidemiology Clin Microbiol Infect 2007 13 Suppl 3 1 46 17716294 10.1111/j.1469-0691.2007.01786.x

[B24] Bartual SG Seifert H Hippler C Luzon MA Wisplinghoff H Rodriguez-Valera F Development of a multilocus sequence typing scheme for characterization of clinical isolates of *Acinetobacter baumannii* J Clin Microbiol 2005 43 4382 4390 16145081 10.1128/JCM.43.9.4382-4390.2005 PMC1234098

[B25] Hamidian M Hall RM AbaR4 replaces AbaR3 in a carbapenem-resistant *Acinetobacter baumannii* isolate belonging to global clone 1 from an Australian hospital J Antimicrob Chemother 2011 66 2484 2491 21873287 10.1093/jac/dkr356

[B26] Diancourt L Passet V Nemec A Dijkshoorn L Brisse S The population structure of *Acinetobacter baumannii*: expanding multiresistant clones from an ancestral susceptible genetic pool PLoS One 2010 5 e10034 20383326 10.1371/journal.pone.0010034 PMC2850921

